# Impact of Powered Knee-Ankle Prosthesis on Low Back Muscle Mechanics in Transfemoral Amputees: A Case Series

**DOI:** 10.3389/fnins.2018.00134

**Published:** 2018-03-22

**Authors:** Chandrasekaran Jayaraman, Shenan Hoppe-Ludwig, Susan Deems-Dluhy, Matt McGuire, Chaithanya Mummidisetty, Rachel Siegal, Aileen Naef, Brian E. Lawson, Michael Goldfarb, Keith E. Gordon, Arun Jayaraman

**Affiliations:** ^1^Max Nader Lab for Rehabilitation Technologies & Outcomes Research, Center for Bionic Medicine, Shirley Ryan Ability Lab, Chicago, IL, United States; ^2^Department of Physical Therapy and Human Movement Sciences, Northwestern University, Chicago, IL, United States; ^3^School of Life Sciences, Swiss Federal Institute of Technology in Lausanne, Lausanne, Switzerland; ^4^Department of Mechanical Engineering, Vanderbilt University, Nashville, TN, United States

**Keywords:** powered knee-ankle prosthesis, amputees, gait, variability, musculoskeletal injuries, microprocessor knee, low back pain

## Abstract

Regular use of prostheses is critical for individuals with lower limb amputations to achieve everyday mobility, maintain physical and physiological health, and achieve a better quality of life. Use of prostheses is influenced by numerous factors, with prosthetic design playing a critical role in facilitating mobility for an amputee. Thus, prostheses design can either promote biomechanically efficient or inefficient gait behavior. In addition to increased energy expenditure, inefficient gait behavior can expose prosthetic user to an increased risk of secondary musculoskeletal injuries and may eventually lead to rejection of the prosthesis. Consequently, researchers have utilized the technological advancements in various fields to improve prosthetic devices and customize them for user specific needs. One evolving technology is powered prosthetic components. Presently, an active area in lower limb prosthetic research is the design of novel controllers and components in order to enable the users of such powered devices to be able to reproduce gait biomechanics that are similar in behavior to a healthy limb. In this case series, we studied the impact of using a powered knee-ankle prostheses (PKA) on two transfemoral amputees who currently use advanced microprocessor controlled knee prostheses (MPK). We utilized outcomes pertaining to kinematics, kinetics, metabolics, and functional activities of daily living to compare the efficacy between the MPK and PKA devices. Our results suggests that the PKA allows the participants to walk with gait kinematics similar to normal gait patterns observed in a healthy limb. Additionally, it was observed that use of the PKA reduced the level of asymmetry in terms of mechanical loading and muscle activation, specifically in the low back spinae regions and lower extremity muscles. Further, the PKA allowed the participants to achieve a greater range of cadence than their predicate MPK, thus allowing them to safely ambulate in variable environments and dynamically control speed changes. Based on the results of this case series, it appears that there is considerable potential for powered prosthetic components to provide safe and efficient gait for individuals with above the knee amputation.

## Introduction & background

Prostheses are defined as devices that help to restore a missing function that has occurred as the result of limb loss. While many factors affect an amputee's ability to return to their pre-amputation functional level, the design of the prosthetic device itself can impact function by contributing to normalization of gait and body symmetry. In the United States, prescription of a specific type of device for a user depends on the type of amputation and their clinically designated mobility level [i.e., K levels or Medicare Functional Classifications Level (MFCL); CMS, 2001].

Consequently, various types of prosthetic device designs have evolved over decades, with the goal of facilitating normative (reproducing healthy limb behavior) biomechanical gait behavior in amputees. Broadly, there are three main categories of prosthetic knee and ankle components available for transfemoral (above knee) amputees when considering energetic control, namely, (i) mechanical passive devices (non-powered) (Michael, 1999), (ii) microprocessor-controlled passive devices (Grimes et al., 1977; Peeraer et al., 1989, 1990; Aeyels et al., 1992; Popović et al., 1995; Taylor et al., 1996; Otto Bock Orthopedic Industry, 1998; Zlatnik et al., 2002; Ossur, 2017), and (iii) powered devices (Tomovic and McGhee, 1966; Flowers, 1974; Au et al., 2008, 2009; Sup et al., 2009; Hitt et al., 2010). The powered devices for the transfemoral population can be further divided into powered knees, powered ankles, and powered knee-ankle devices (Cappozzo and Gazzani, 1982; Au et al., 2008, 2009; Holgate et al., 2008; Bergelin et al., 2010; Eilenberg et al., 2010; Hitt et al., 2010; Suzuki et al., 2011; Bergelin and Voglewede, 2012; Caputo and Collins, 2014; Cherelle et al., 2014).

Walking using traditional non-powered prostheses is very energy inefficient (incurring ~60% more energy usage) when compared to able-bodied individuals resulting in reduced everyday mobility or even immobility (Hafner et al., 2002). Additionally, transfemoral amputees commonly exhibit compensatory biomechanics resulting in body motions that are atypical to normal human locomotion. These compensatory mechanisms arise due to chronic imbalance or prosthetic derived muscle/movement activations that alter the normal biomechanics and motion. Over time, these factors increase the risk of secondary musculoskeletal injuries such as severe chronic pain in the low back and the contralateral (non-amputated) side resulting in inactivity or surgical interventions (Cappozzo and Gazzani, 1982; Michaud et al., 2000; Klein Horsman et al., 2007; Goujon-Pillet et al., 2008; Molina Rueda et al., 2013; Devan et al., 2014; Hendershot and Wolf, 2014; Shojaei et al., 2016). Therefore, any enhancement to the mechanical or control systems design of prostheses which can reproduce a biomechanical behavior similar to a healthy limb is very beneficial.

In the pursuit of normalizing some of the abnormal gait mechanics that are commonly seen in transfemoral amputees, a coordinated powered knee and ankle prosthesis (Generation 3) was developed at Vanderbilt University to provide power generation similar to an anatomical joint. While there are commercially available, independent prosthetic knees and feet that provide power to a single joint, there are no available versions that have integrated power and communication between both the knee and ankle components. Thus, implementation of the PKA in transfemoral participants has the potential to improve lower limb prosthesis performance.

Congruent with this tenet, literature indicates that the Vanderbilt Generation 3 powered knee-ankle prosthesis (PKA) may provide significant biomechanical benefits to users, compared to conventional passive devices (Goldfarb et al., 2013; Lawson et al., 2013, 2014, 2015; Shultz et al., 2016). Furthermore, most lower-limb amputation studies have historically focused on comparing the performance of a traditional mechanically passive prostheses to microprocessor-controlled knee prostheses (MPK) with variable damping. These studies suggest that in comparison to mechanical passive devices, consistent use of MPK prostheses reduced energy consumption, improved smoothness of gait, and decreased the work done by the affected side hip muscles during walking (Taylor et al., 1996; Schmalz et al., 2002; Johansson et al., 2005).

However, presently it is not clear if implementing a PKA in unilateral transfemoral amputees that currently use a microprocessor controlled knee (MPK) could offer improved biomechanical benefits. Such biomechanical benefits, if any, could pave the way for them to reproduce a normalized gait similar to the healthy limb in comparison to their predicate MPK device. Additionally improving body biomechanics in transfemoral amputees could potentially minimize the risk of exposure of the low back region and contralateral side, to abnormal loading-based secondary musculoskeletal injuries in transfemoral amputees (a serious health-concern in transfemoral amputees; Devan et al., 2017). Consequently, this case series investigated the potential benefits the PKA could offer to transfemoral amputees who are currently using a MPK as their predicate device. To achieve this, a clinical comparison of the performance between the PKA and the participants predicate MPK devices was conducted in two transfemoral amputees.

Through this case series we hope to provide two novel insights. It is the first to report a performance comparison between the PKA and MPK prosthetic device in unilateral transfemoral amputee literature. Secondly, this case series compared the low back (L3 lumbar erector spinae region) muscle activation in unilateral transfemoral amputees ambulating with the PKA and their MPK. Low back muscle activation and injury has been very scarcely studied in transfemoral prosthetic literature (Yoder et al., 2015; Shojaei et al., 2016). The novel information from this case series will provide novel insights that can aid in improving our understanding on potential benefits the PKA could offer over the MPK devices. In terms of low back muscle loading pattern (i.e., reduce activation asymmetry in the contralateral vs. ipsilateral side).

## Methods

### Ethics

All study procedures were approved by the Institutional Review Board at Northwestern University. Both participants provided voluntary signed informed consent before beginning the study.

The cases discussed here are part of a larger clinical trial that can be found at https://clinicaltrials.gov/ct2/show/NCT03204513.

### Case description

The basic demographic information and prosthetic device specifications of the two study participants are provided in Table S1 (Supplementary Material).

Even though both study participants utilize a MPK prosthesis, they have clinical differences based on age, residual limb length, clinically perceived activity level, and everyday community mobility. CS01 is a 25 y/o male with a knee disarticulation amputation, who currently has a clinically identified functional level of MFCL K4, indicating that he “has the ability [or potential] for prosthetic ambulation that exceeds basic ambulation skills, exhibiting high impact, stress or energy levels which is typical of an active adult or athlete” (CMS, 2001). CS01 is a student athlete, who plays basketball 3–5 times a week. CS02 is a 58 y/o male with a medium length transfemoral amputation whose current MFCL level is K3, indicating that he “has the ability [or potential] for ambulation with variable cadence. Typical of the community ambulators who have the ability to traverse most environmental barriers and may have vocational, therapeutic or exercise activity that demands prosthetic utilization beyond simple locomotion” (CMS, 2001). CS02 is employed as a computer engineer. His personal life includes maintenance of a large piece of land and care of multiple large breed dogs. He has already had a total knee replacement of the intact limb. The differences in the lengths of the residual limbs can play an important factor in the control of a prosthesis. The shorter the residual limb, the less control a participant would have due to loss of muscle, nerve and bony lever arm. Significantly, CS01's level of amputation, a knee disarticulation, provides almost fully intact hip adductors and the vast majority of the major muscle group's bulk remaining. This is not the case in CS02's mid length amputation, transecting all of the major muscle groups of the thigh, reducing his capacity to generate force. In general, a shorter residuum means additional work of the smaller remaining muscles with less biomechanical advantage. Over time, these imbalances can cause compensations in other areas of the body. Additionally, a longer residual limb may require differences in the prosthetic knee height compared to the anatomical knee axis, which may contribute to inequalities in gait kinematics.

On participants' similarities at the time of the study, both had been using their current MPK devices for over 2 years. Both subjects demonstrated reduced hip extension compared to normative range of motion, though they were both able to achieve functional hip range of motion through compensatory motions of the lumbar spine. When a hip flexion contracture is present, the step length of the sound limb is restricted as well. This impacts a participant's overall gait, including the quality of steps, speed and distance covered. In order to accommodate the hip flexion contracture, motion usually occurs within the spine.

Photographs of both participants with their predicate prosthetic device and the PKA have been provided in the Supplementary Material (Figures S1, S2).

### Study design

Participants were randomized to start either with their predicate MPK-1 (Genium in case of participant CS01), and MPK-2 (Rheo-3 in case of participant CS02), or the PKA. Following the consent process, an experienced prosthetist evaluated the participants' prosthetic sockets for appropriate component fitting with the study device (PKA) or predicate device (MPK-1 for CS01 and MPK-2 for CS02). Any adjustments to the sockets or the device settings were made during the fitting sessions.

#### Prosthetic device fitting

The knee and ankle parameters for the PKA were individually configured for each participant during the fitting sessions. In brief, the impedance parameters at the ankle and knee joints during three states (sitting, standing, and stepping), and the push-off trigger angle and push-off strength thresholds were manually tuned starting with reference parameters used from the data of healthy individuals (Sup et al., 2008). The ankle motor power to enable ankle push-off strength was also individually adjusted to suit the participants comfort level for the three cadence levels (slow, default, and fast speeds).

Additionally, qualitative feedback from the participant and external observation by the clinicians were used to fine tune the parameters so that any undesirable aspects of gait arising from compensations, such as vaulting, hip hiking, and circumduction were minimized. The goal of the tuning process was to adjust the PKA device to maximize participant's ability to ambulate with a biomechanical behavior similar to a healthy limb. The overall process of tuning the PKA device for a participant is similar to the process followed by prosthetist in aligning and adjusting any passive or powered prosthetic device. The procedure for this customized parameter tuning for the PKA has been discussed in extensive detail in literature (Lawson et al., 2013, 2014, 2015; Shultz et al., 2016). The finalized parameters for the two participants are provided in the Supplementary Material (Table S2, Figures S3, S4).

#### Prosthetic device training

Once proper prosthetic fit was clinically confirmed, participants underwent up to 12 training sessions of intense functional training with the device. Subject's body mechanics were evaluated and training was provided to maximize control of the prostheses and minimize compensations. The training included performing a battery of activities indoors and outdoors, and emulating walking environments encountered in daily living conditions (e.g., level and uneven indoor and outdoor surfaces including obstacle avoidance, crossing streets, and varied pavement). Participants were also specifically acclimated to treadmill walking. Safe and independent performance of the benchmark activities over three of the training sessions was used as a threshold to indicate the successful completion of training with the device (Supplementary Material, Table S3). These training procedures were performed with the participant's predicate devices (MPK-1 for CS01 and MPK-2 for CS02), as well as the PKA. Training for both the devices was carried out to maximize device usage in the training environment and minimize any confounding effects arising due to the training protocol adopted.

Once the fitting, training, and testing phases for the first study device were completed, a washout period of at least 2 months was given before the participant was scheduled to cross-over to the second device. This was done to minimize the carry-over effect of one device influencing the performance outcomes of the second device. The order of the post-training assessment tests for both devices were held similar for each participant.

### Data collection procedures

Three different strands of tests were conducted to compare the performance between MPKs and the PKA. Ankle and knee kinematics were analyzed to investigate if using a PKA enabled participants to emulate an ankle and knee behavior similar in biomechanics to the behavior of a healthy limb. Muscle activation was recorded from the lower limb muscles on the contralateral side (non-amputated side) and the low back lumbar L3 region(bilateral). The muscle activation during ambulation was compared to study the muscle loading trends between the prosthetic devices. A modified Graded Treadmill Test (GTT) was performed to investigate the energy efficiency and the ranges of variable cadence the participants achieved with these devices. Finally, to assess the prosthetic devices on a functional task, an outdoor cross-walk test was performed to represent a common activity of daily living. Participants performed all the tests with both the devices.

#### Biomechanics

A 10 camera motion capture system (Qualysis, Gothenburg Sweden) was used to record the kinematics, ground reaction forces (GRF), and muscle activation (EMG) during walking. In total, 38 reflective markers were placed on the lower limbs, pelvis, and trunk based on the six degrees of freedom cluster marker configuration (Acasio et al., 2017) The motion capture data was sampled at 100 Hz. GRF were collected using six AMTI force plates (AMTI, Watertown, MA) sampled at 1,000 Hz. Muscle activation using wireless EMG sensors (Delsys Inc.) were collected from bilateral erector spinae muscles at the lumbar L3 level (RES-L3 and LES-L3) and on lower extremity muscles [medial gastrocnemius (MGC), rectus femoris (RF)] on the limb contralateral to the prosthesis. The EMG's were sampled at 2,000 Hz. All data was collected during walking at the participants self-selected speed along a seven-foot walkway embedded with force plates.

#### Modified graded treadmill test (VO2 and variable cadence)

This test was used to determine each participant's cardio-vascular response to walking at different speeds on a motorized treadmill. Participants were secured to an overhead safety harness during this test as they walked for up to 2 min at progressively increasing speeds on the treadmill. Speeds were varied between 0.2 m/s up to 2.0 m/s at increments of 0.2 m/s. Before increasing the speed to the next stage, participants were given the choice to stop or continue with the test. The test was stopped if the participant opted to do so, or if the clinician decided to stop the test based on achieving age-based target maximum heart rate threshold. The maximum heart rate threshold was calculated as 80% of their maximum heart rate (220-Age). Participants' cardiovascular and metabolic responses (Duffield et al., 2004) were monitored frequently and recorded during the entire test using a COSMED K4B2 device (Duffield et al., 2004). Additionally, inertial measurement units (IMUs–Actigraph GT9X Link, Actigraph, LLC. Pensacola, FL, USA.; Rothney et al., 2008; John and Freedson, 2012), were mounted bilaterally on the dorsum of the shoes to capture acceleration signatures during walking. This was post-processed to extract cadence (step counts/min) and stride times.

#### Outdoor overground walking (EMG)—MC10

Participants performed a cross walk blinking signal test. This test measured the time taken to cross a designated two-lane street with curb cuts at the transition to the sidewalk. The walkway distance was ~20 m. The muscle activation of the MGC was also recorded from the contralateral limb using the BioStampRC, a novel high resolution skin conformable flexible Bluetooth based sensor (Yuhao et al., 2018). The EMG module sampled at 1,000 Hz while the acceleration modules sampled at 31.25 Hz. Both EMG and acceleration were recorded simultaneously during the task. Participants performed three trials.

## Data analysis

Standard data analysis procedures were employed to post-process the data and extract the outcome metrics of interest. Custom developed MATLAB scripts were used for all data analysis. All the outcome metrics were computed and compared between the devices (i.e., PKA and the respective predicate MPK devices for CS01 and CS02) to study various aspects within the context to performance, safety (potential to minimize injury) and function.

### Healthy controls

To compare the prosthetic device performance for knee and ankle kinematics, the healthy benchmark data from literature was used (Winter, 1991). This approach is a commonly adopted procedure in prosthetic literature in order to compare biomechanical behavior (Goldfarb et al., 2013; Lawson et al., 2013, 2014, 2015; Shultz et al., 2016).

### Biomechanics

All motion capture data was post-processed using Visual3D (C-Motion, Germantown, MD) and custom MATLAB (version R2016, Mathworks, Natick, MA) scripts. Any missing marker data was gap-filled and low-pass filtered (Butterworth, cut-off frequency 6 Hz). GRF's were low-pass filtered (Butterworth, cut-off frequency 20 Hz). The gait cycle was identified based on the heel strike events from motion data. Each gait cycle was then normalized from 0 to 100%. In total, six strides were analyzed. All data was averaged over three walking trials (i.e., six steady state strides).

#### Joint kinematics

An inverse kinematics pipeline was executed in Visual 3D to compute the ankle and knee joint kinematics from the motion capture data. The knee and the ankle joint kinematics from each of the devices were then benchmarked with the knee and ankle joint kinematics from the healthy controls data obtained from literature (Winter, 1991). Pearson correlation coefficients were then computed between the joint kinematics obtained from each of the prosthetic devices and that of the healthy controls from literature (Winter, 1991). The strength of this correlation (positive correlation value between 0 and 1) indicated the degree of closeness of a particular device to reproduce kinematic behavior similar to a healthy limb. A correlation value of zero indicates that the knee/ankle kinematics trajectory behavior while using that prosthetic device did not linearly correlate (temporally) with the joint kinematic behavior of a healthy limb as obtained from literature (Winter, 1991).

#### Vertical ground reaction force (VGRF)

The vertical ground reaction force (VGRF) from the contralateral and ipsilateral sides was extracted from the force plate recordings. The VGRF was then averaged over the gait cycles to obtain the mean VGRF, and then normalized using the participant's weight (participant+device) in kilograms (i.e., N/kg). The ratio of, the *peak weight- normalized VGRF following the heel strike* (F_Z1_ N/kg), *over the peak weight-normalized VGRF at ankle push-off* (F_Z2_ N/kg) *during stance phase was computed* (refer schematic in Figure S5). A value of unity for this ratio indicates that the peak VGRF during these two instances of stance phase were of equal magnitude. A deviation of this ratio from unity marks the degree of asymmetry between the peak VGRF during stance phase of gait. The PKA provides push off power at the ankle during the terminal stance phase of the gait unlike the MPK. In order to study the benefit of the ankle push off in normalizing the VGRF peaks, this ratio measure was chosen. This ratio from both the prosthetic devices (MPKs and PKA) was then benchmarked with the ratio of VGRF obtained for the healthy controls data from literature (Winter, 1991).

#### EMG data analysis

The EMG data was band pass filtered (Butterworth, band pass frequency 30–500 Hz). From each of the three walking trials, two steady state gait cycles were extracted bilaterally. The cycle wise gait data was extracted based on the heel strike events. Each gait cycle was then normalized from 0 to 100%. The area under the curve (AUC) was then computed for each muscle group from each of the gait cycle. This value was then averaged over the gait cycles and averaged over the trials for each side. This was computed for the right and left erector spinae at lumbar L3 level (RES-L3 and LES-L3), MGC and RF of the contralateral limb. In order to study the symmetry of lumbar muscle activation between the contralateral and ipsilateral sides during the overall gait cycle, the ratio of the AUC between both these sides was computed. A ratio closer to unity indicates overall symmetric EMG activation (i.e., in terms of gross magnitude of AUC) bilaterally on the erector spinae muscles at the L3 level.

### Clinical recordings

The first minute in each speed during the GTT was used to attain the steady state locomotion and hence was not used for data analysis. All outcome metrics for the GTT were computed for the second minute of each speed during the GTT.

#### COSMED

Manufacturer provided proprietary software was used to extract the VO2 and energy expenditure from the COSMED K4B2 during the modified GTT. The VO2 and energy expenditure were computed for the second minute of each speed during the GTT. The energy expenditure was used to study the energy efficiency of the PKA vs. the predicate MPK devices. The metabolic outcomes were weight normalized (participant weight + device weight). The gross oxygen cost (mL/kg/m) [i.e., from every second minute (steady state) over the entire trial] was also computed to use as an overall index to compare the gait efficiency while ambulating with different prosthetic devices.

#### IMU's

The vertical acceleration (A_y_) from the IMUs placed on the dorsum of each foot was used to compute the cadence (step count/min) and stride time during the modified GTT. The data from the second minute of each time series was extracted based on the IMU time stamp. Then a continuous wavelet transform was used on the A_y_ component to compute the stride times and step counts (Zijlstra and Hof, 2003). At each speed range, the mean, standard deviation (*SD*), and the coefficient of variation [CV% = (SD/mean) ^*^100] were computed for the stride time data. Custom developed MATLAB scripts were used for all IMU data post-processing.

## Results

The results are presented in three sections (i) biomechanics, (ii) clinical outcomes from GTT, and (iii) functional outcomes from outdoor testing. Both participants completed the protocol within the described timeline and there were no adverse events.

### PKA parameter tuning

The participant-wise final parameters set for the PKA devices are provided in Table S2 in the Supplementary Material. Participant CS01 preferred a lower strength for the ankle push off power from the PKA device in comparison to CS02 (Table S2, Figures S3, S4 from Supplementary Material). The ankle reference trajectories (Figures S3, S4) were adjusted for each participant in order to allow them to clear the foot during swing phase while walking at various speeds. During the swing phase of the gait, the controller of the PKA is designed such that the knee and the ankle followed the reference trajectories.

### Biomechanics

The mean values of the temporal spatial variables, including walking speed, stride time, and stance time (% of gait cycle) when walking with the MPKs and PKA are provided in the Table 1. The mean speed, mean stride time, and mean stance time (% gait cycle) were similar between both the devices for the participants.

**Table 1:** Temporal spatial parameters.

**Gait parameter**	**CS01**		**CS02**	
	**MPK-1 (Genium)**	**PKA**	**MPK-2 (Rheo-3)**	**PKA**
Walking speed (m/s)	1.3 (0.08)	1.3 (0.11)	1.2 (0.11)	1.3 (0.07)
Intact leg stride time (s)	1.1 (0.02)	1.1 (0.03)	1.2 (0.05)	1.2 (0.05)
Prosthetic leg stride time (s)	1.1 (0.02)	1.1 (0.05)	1.2 (.03)	1.2 (0.05)
Intact leg stance time (% gait cycle)	65 (1.1)	65 (4.0)	70 (4.0)	71 (2.0)
Prosthetic leg stance time (% gait cycle)	62 (1.1)	64 (2.0)	61 (2.0)	60 (2.0)
Intact leg stance phase: Ratio of VGRF (F_Z1_/F_Z2_)	1.18 (0.1)	1.07 (0.03)	1.13 (0.11)	1.01 (0.03)
Prosthetic leg stance phase: Ratio of VGRF (F_Z1_/F_Z2_)	1.17 (0.2)	1.00 (0.03)	1.12 (0.02)	1.00(.02)

#### Joint kinematics

**Figure 1:**
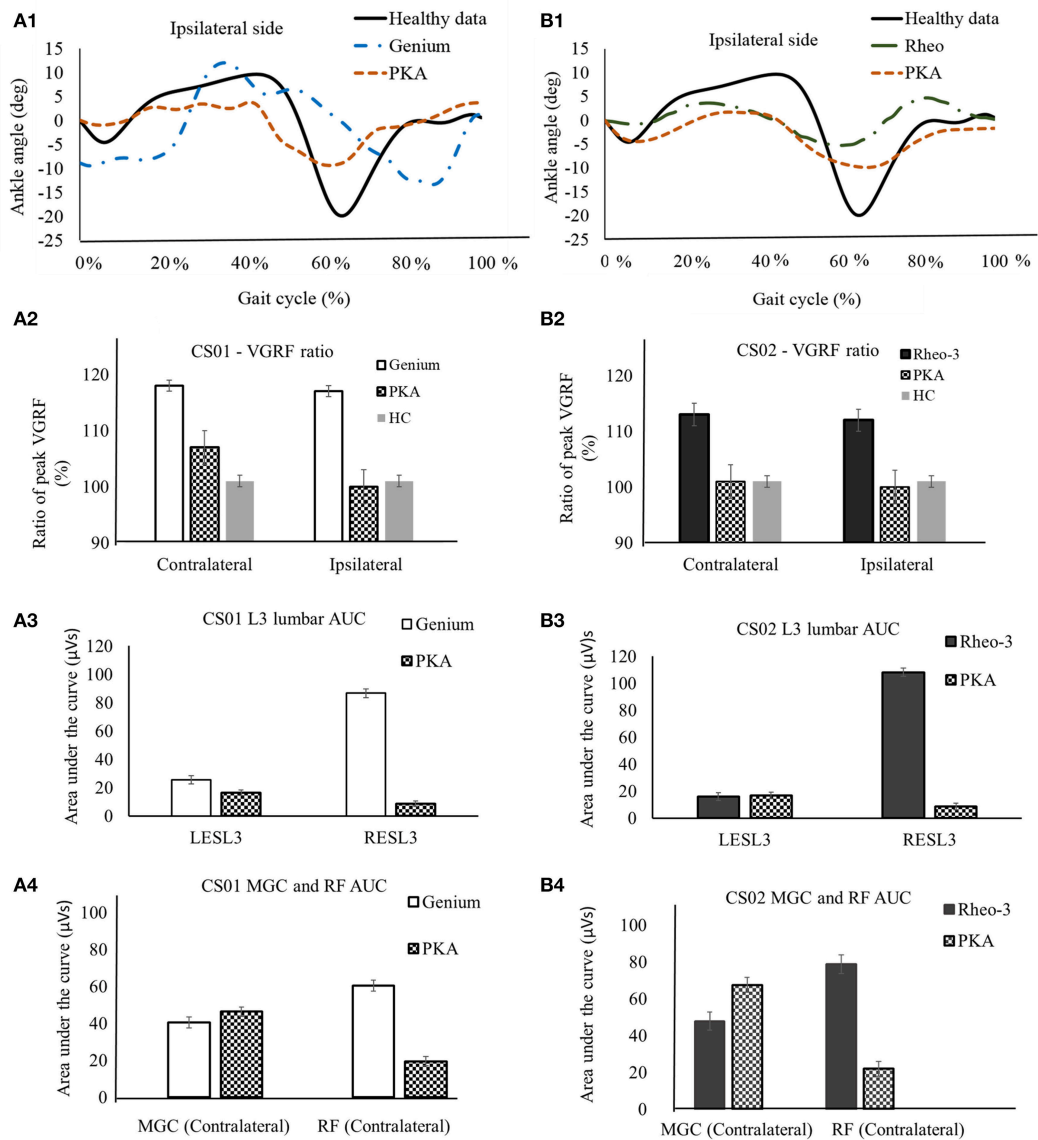
A representative data showing comparison of biomechanics outcome metrics between MPK and PKA. Panels **(A1–A4)** are for participant CS01. **(A1)** compares the ankle joint kinematics between PKA, MPK1- (Genium), and healthy data (Winter, 1991) for the ipsilateral side for CS01. **(A2)** Shows comparison of the ratio of peak VGRF (Fz1/Fz2) during the stance phase between MPK-1 (Genium), PKA, and healthy data (Winter, 1991). It was observed that using the PKA led to a VGRF behavior closer to healthy limb on both the contralateral and the ipsilateral sides. **(A3)** EMG activation profile between MPK-1(Genium) and PKA at right and left side L3 erector spinae muscles in low back. The EMG activation profile was indexed as area under the curve (AUC) of the EMG signal. It can be observed that using PKA reduces the asymmetry in EMG activation between the LES-L3 and the RES-L3 muscles. **(A4)** EMG activation (AUC) for lower extremity muscles MGC and RF on contralateral side. It was observed that using the PKA lead to higher activation in the MGC and reduced activation in the RF on the contralateral side. Panels **(B1–B4)** are for participant CS02. **(B1)** compares the ankle joint kinematics between PKA, MPK2- Rheo-3, and healthy data (Winter, 1991) for the ipsilateral side for CS02. **(B2)** Comparison of the ratio of peak VGRFs during the stance phase between MPK-2 (Rheo-3), PKA, and healthy data (Winter, 1991). It was observed that using the PKA led to a VGRF behavior close to healthy limb on both, the contralateral and the ipsilateral sides. **(B3)** EMG activation profile between MPK-2 (Rheo-3) and PKA at right and left side L3 erector spinae muscles in low back. The EMG activation profile was indexed as area under the curve (AUC) of the EMG signal. It can be observed that using PKA reduces the asymmetry in EMG activation between the LESL3 and the RESL3 muscles. **(B4)** EMG activation (AUC) for lower extremity muscles MGC and the RF on contralateral side. It was observed that using the PKA lead to higher activation in the MGC and reduced activation in the RF on the contralateral side.

Figures 1A1,B1 shows a representative plot comparing the ankle joint kinematics for the two participants (CS01 and CS02) while using their predicate MPK devices and the PKA. The ankle joint kinematics while using PKA (Pearson's correlation coefficient: rho ≥ 0.6) were closer in kinematics to a healthy limb gait behavior than the MPK devices (correlation coefficient: rho ≤ 0.3). A similar observation was noted for knee kinematics when using the PKA (Pearson's correlation coefficient: PKA: rho _PKA_ ≥ 0.95; MPKs: 0.92 ≤rho _MPKs_ ≤ 0.95).This observation is consistent with the literature (Sup et al., 2009; Lawson et al., 2013, 2015). This showed that both the participants reproduced ankle and knee kinematics closer to that observed in healthy limb while ambulating using the PKA. Similarity in kinematics to a healthy limb have been shown to lead to symmetric joint loading profile during gait (Sup et al., 2009; Lawson et al., 2013, 2015). To further understand the implication, here we study two aspects, (i) the symmetry in ratio of peak VGRF (i.e., F_Z1_/F_Z2_) and, (ii) the overall low back muscle activation (erector spinae at lumbar L3 EMG AUC) while walking with the predicate MPKs and the PKA.

#### Ratio of peak VGRF (F_Z1_/F_Z2_)

The ratio of the peak *VGRF* from the contralateral and the ipsilateral sides are furnished in Table 1. The ratio of peak VGRF between the contralateral and the ipsilateral side while using the different devices are compared with the healthy control in Figures 1A2,B2. Both participants had at least a 12% difference between the magnitude of the ratio of the peak VGRF (VGRF _FZ1_ > VGRF F_Z2_, varied between 12 and 18%, Table 1). In comparison to the MPK, this ratio was lower (<7% difference) while using the PKA in both participants (Table 1, Figures 1A2,B2). The peak VGRF magnitudes encountered while walking with both the MPKs and PKA fell well within the standard norm (i.e., 100–120% of body weight). However, based on the *ratio of the VGRF*, both participants reproduced behavior more similar to healthy controls when using the PKA.

#### EMG activation (AUC)

In comparison to the MPKs, the erector spinae muscle activation (LES-L3 and RES-L3) was relatively more symmetrical (magnitude of AUC) between the contralateral and ipsilateral sides while using the PKA (Figures 1A3,B3). However, in both participants using the MPK, the activation of the RES-L3 was approximately five times higher than that of the LES-L3 (Figures 1A3,B3), implying a degree of asymmetry in muscle activation while using the MPK. In contrast, while using the PKA, this asymmetry was far less pronounced. Complimenting this observation, it was observed that the muscle activation (AUC) of the RF on the contralateral side decreased considerably while using the PKA in both participants. The MGC activation on the contralateral lateral side increased for CS02 while using the PKA (Figure 1B4). However, for participant CS01, the MGC showed only a slight increase in activation in contralateral side when using the PKA (Figure 1A4).

### Clinical outcomes (GTT)

**Figure 2:**
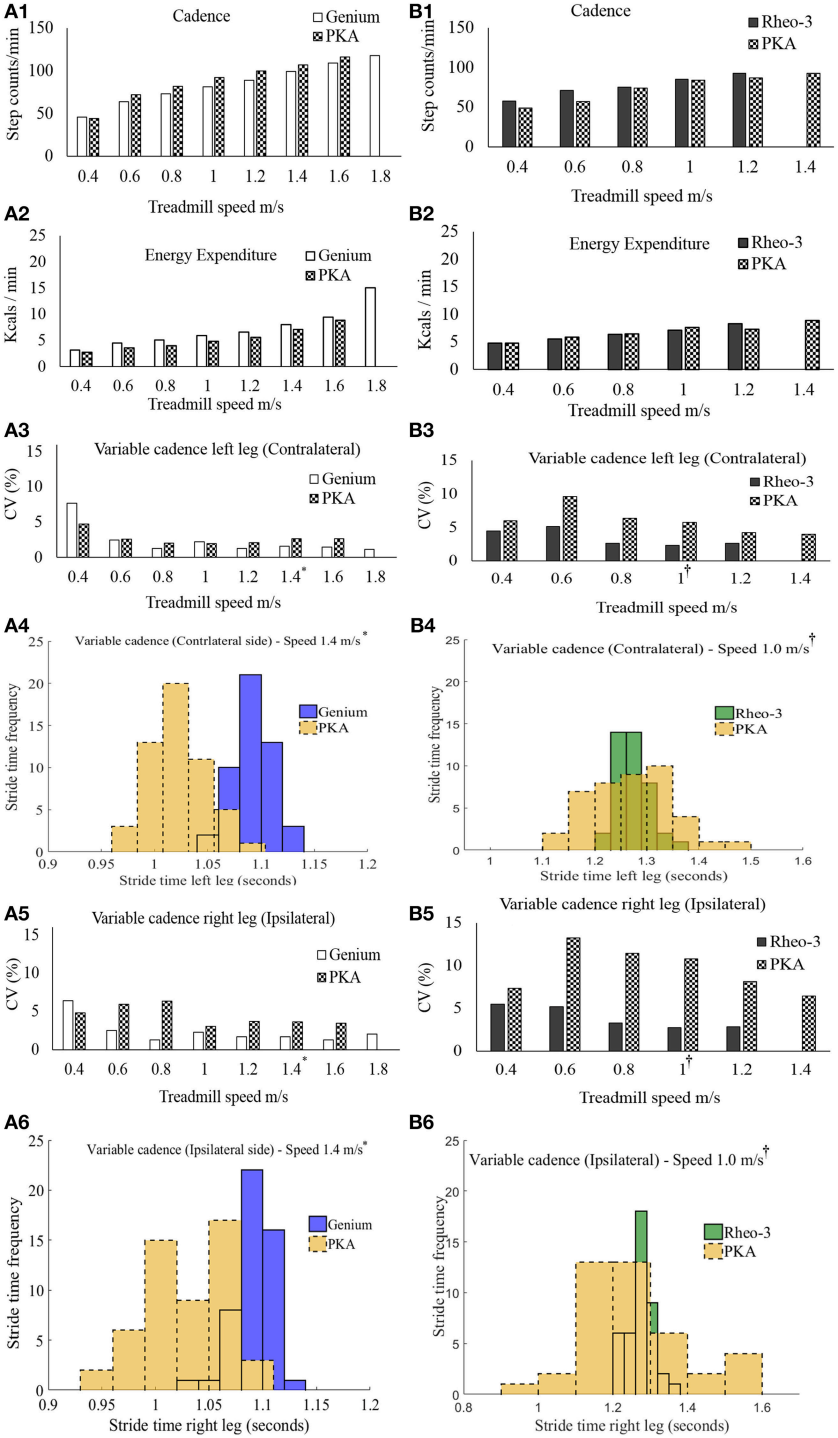
Summary of outcome metrics from the modified graded treadmill test (GTT). Panels **(A1–A6)** are GTT outcome metrics for participant CS01 and panels **(B1–B6)** are GTT outcome metrics for participant CS02. **(A1,B1)** compares the cadence during the GTT between the MPK-1(Genium) vs. PKA and MPK-2(Rheo-3) vs. PKA respectively. **(A2,B2)** compares the EE during GTT between the MPK-1 (Genium) vs. PKA and MPK-2 (Rheo-3) vs. PKA respectively. **(A3,B3)** Compares the coefficient of variation (CV%) for the stride time between the MPK-1(Genium) vs. PKA (in panel **A3**) and MPK-2(Rheo-3) vs. PKA (in panel **B3**) respectively for the contralateral limb. **(A4,B4)** shows the stepwise stride time for the contralateral side during a representative 1 min treadmill walk for MPK-1(Genium) vs. PKA [(in panel **A4**): at treadmill speed of 1.4 m/s (^*^preferred treadmill speed of the participant CS01)] and MPK-2(Rheo-3) vs. PKA [(in panel **B4**): at treadmill speed of 1 m/s (^†^preferred treadmill speed of the participant CS02)]. **(A5,B5)** Compares the coefficient of variation (CV%) for the stride time between the MPK-1(Genium) vs. PKA (in panel **A5**) and MPK-2(Rheo-3) vs. PKA (in panel **B5**) respectively for the ipsilateral limb. **(A6,B6)** shows the stepwise stride time for the contralateral side during a representative 1 min treadmill walk for MPK-1(Genium) vs. PKA [(in panel **A6**): at treadmill speed of 1.4 m/s] and MPK-2(Rheo-3) vs. PKA [(in panel **B6**): at treadmill speed of 1 m/s].

The summary of the GTT results are shown in Figure 2.

#### Speed ranges

Participant CS01 was able to reach a maximum speed of 1.8 m/s while using the MPK-1 and reached a maximum speed of 1.6 m/s while using the PKA. Participant CS01 transitioned from walking into running when the speed was switched from 1.6 to 1.8 m/s while using MPK-1. In contrast, participant CS02 was able to reach a higher walking speed of 1.4 m/s while using the PKA compared to a maximum speed of 1.2 m/s with the MPK-2.

#### Energy expenditure (EE)

The overall energy expenditure (EE) trends during the GTT task showed a marginal benefit using the PKA in comparison to the MPK for participant CS01. For participant CS02 energy benefits were observed at certain speed ranges while using the MPK-2 (Figure 2B2). These comparisons are based on the minute-by-minute outcomes and for matched gait speed. Moreover, despite being approximately twice as heavy as the predicate MPKs' weight, the PKA did not require additional energy expenditure during the GTT task (Figures 2A2,B2). In addition to looking at the minute-to-minute EE, a gross measure of the overall oxygen cost (i.e., gait efficiency; Darter et al., 2013) was also calculated. Based on the oxygen cost the gross gait efficiency during the entire GTT test was as follows, CS01 [(MPK-1 _GTT_ = 0.16 mL/kg/m); PKA _GTT_ = 0.14 mL/Kg/m)], CS02 [(MPK-2 _GTT_ = 0.22 mL/kg/m); PKA _GTT_ = 0.22 mL/Kg/m)]. Based on the gait efficiency, using the PKA was more energy efficient for CS01 and incurred the same energy cost as the MPK-2 for CS02.

#### Variable cadence

The stride times computed from the foot IMUs were used to compare the ranges of variable cadence achievable between the PKA and MPK during the modified GTT. It was observed that at all speed levels during the GTT, participants were able to walk with variable cadence while using both prostheses (i.e., their respective predicate MPK-1, or MPK-2 and the PKA; Figures 2A1,B1). However, at speed ranges >0.6 m/s, the variability in stride times indexed as the coefficient of variation (CV%) was relatively higher for the PKA for both users bilaterally. This suggests that the PKA offered greater ranges of variable cadence to both the contralateral (Figures 2A3,B3,A4,B4) and the ipsilateral side limbs (Figures 2A5,B5,A6,B6). Perhaps the ability of the PKA device to offer, (i) greater range of variable cadence and, (ii) ankle push off, facilitated marginally better performance in terms of EE/endurance. Indeed, literature shows that cadence and energy expenditure are positively correlated in lower limb amputee population (Rowe et al., 2014).

### Outdoor

**Figure 3:**
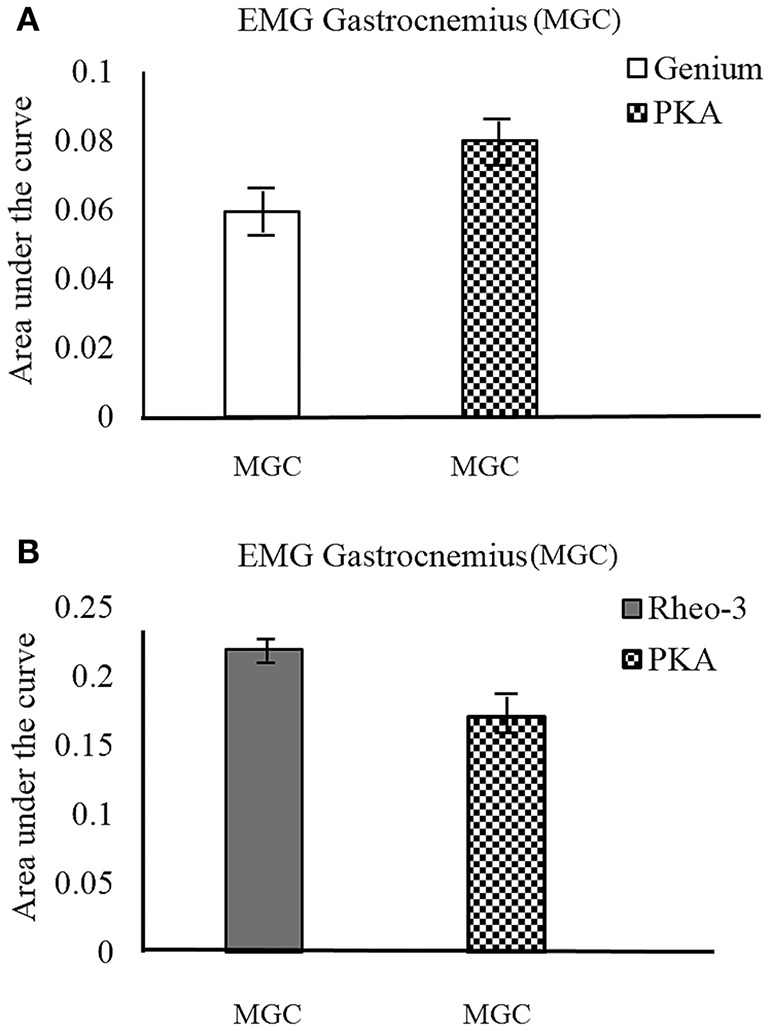
MGC muscle EMG activation profile during outdoor cross walk testing. The EMG activation was indexed as area under the curve (AUC) of the EMG signal. **(A)** compares the MGC muscle activation on the contralateral side between the MPK-1(Genium) and the PKA device. **(B)** compares the MGC muscle activation on the contralateral side between the MPK-2(Rheo-3) and the PKA device for CS02.

The participants were able to cross the street to demonstrate community mobility and speed modulation (walking speeds: CS01_Genium_ = 1.9 m/s, CS01_PKA_ = 1.6 m/s, CS02_Rheo−3_ = 1.6 m/s, CS02_PKA_ = 1.3 m/s). This showed that both the MPKs and PKA can be used to complete this day-to-day functional task. It was observed that for CS01, the MGC activation followed a similar trend between the outdoor and the indoor tests (level ground walking during the indoor motion capture test; Figure 3A). However, for CS02, the trend for activation of MGC showed opposing trends (i.e., the MPK-2 _AUC_ > PKA _AUC_; Figure 3B) between the outdoor walking and indoor walking (level ground walking during the indoor motion capture test). We speculate that this change in trend for CS02 could have been due to environmental factors such as the uneven terrain and the subject's decreased ability to control frontal plane forces in this environment.

## Discussion

In this two-participant case series we investigated the efficacy of the PKA that incorporates both a powered knee and powered ankle (PKA) system. The main aim was to investigate if the PKA could offer potential benefits to users in terms of gait performance, metabolic performance (EE), and back muscle activation which has implications for minimization of risk of low back pain.

### Benchmarking outcome variables with literature

The mean temporal spatial gait parameters (Table 1), cadence ranges and the mean oxygen cost from the two participants in this case series compared well with mean values reported in literature for transfemoral amputees (Jarvis et al., 2017). The observations on knee and ankle kinematics were also consistent with literature (Sup et al., 2009; Lawson et al., 2013, 2015). Benchmarking our outcome metrics with previous literature provides support for the validity of the data collected and the overall findings.

### Performance

#### Energy expenditure and endurance

CS01, a young adult, achieved a faster gait speed with his predicate device (MPK-1, Genium) on the treadmill in comparison to the PKA. The participant transitioned from walking to running with the MPK at a speed of 1.8 m/s. There are three potential reasons thatCS01 was unable to reach higher speed while using the PKA: (i) the long length of CS01's residual limb, (ii) a lower ankle push-off setting chosen by CS01, which limited him from taking full advantage of the PKA's potential benefit (Supplementary Material, Figure S3) in comparison to CS02, and (iii) there was no “run mode controller” on the Gen-3 PKA used for this study and considering the safety of the participant, we terminated the test before the participant could break into running.

CS02, an older adult, was able to walk at a higher speed with the PKA than the MPK-2 (Rheo-3) during the modified GTT test. CS02 chose to have an ankle pulse setting far higher (Supplementary Material, Figure S4) than that of CS01. Based on this, for an older adult the ankle push off offered by the PKA could be beneficial in terms of facilitating higher walking speed/endurance. Higher walking speed/endurance is generally related to higher quality of living in older adults (Studenski et al., 2011; Busch Tde et al., 2015). However, this tenet may not be generalizable (Hafner et al., 2016). Increased speed/endurance may have occurred due to integration of power at the ankle and knee and been facilitated by the variable cadence feature offered by the PKA.

CS02 had nearly similar gross oxygen cost, (i.e., gait efficiency) during walking on the treadmill with both the MPK-2 and the PKA. However, for the same oxygen cost, CS02 was able to achieve a higher speed and walked longer with the PKA. Participant CS01 had a detectable improvement in gait efficiency while using the PKA (expended less oxygen cost). The gait efficiency while walking with the MPK-1 was 0.02 mL/kg/m higher than that of the PKA for CS01. The minimal detectable change (MDC) threshold for a true change in walking performance (gait efficiency) is 0.01 mL/kg/m (Darter et al., 2013). This showed that the participant CS01 benefitted energetically while using the PKA, while CS02 benefitted in terms of endurance while using the PKA.

### Quality of gait biomechanics and safety

#### Kinematics

The ankle joint kinematics while using the PKA reproduced a trajectory that is more similar in behavior [Pearson's correlation (rho ≥ 0.6)] to that of a healthy limb in comparison to the MPK devices. The PKA device achieves this by virtue of two of its main design features. First, the PKA controller is designed to make the knee and the ankle joints follow an enforced reference trajectory that is similar to a healthy limb trajectory during gait (Supplementary Material, Figures S3, S4) and (ii) the ankle motor in the PKA provides ankle push off power to suit the level of cadence. The PKA also has a powered knee, which provides stabilization throughout the stance phase and provides power to propel the leg during swing phase. Both the predicate devices are energetically passive (i.e., the foot spring stores and releases energy during gait cycle), unlike the PKA, which provides additional power through motors. Both the predicate MPKs and the PKA device reproduced a knee trajectory (kinematics) that was similar to a healthy limb. Previous literature has shown similar results for knee and ankle kinematics. However, they compared the PKA device with a mechanical passive prosthetic device (Goldfarb et al., 2013; Lawson et al., 2013, 2014, 2015; Shultz et al., 2016). This is the first work to report such kinematic comparison between the PKA and MPK devices.

#### Erector spinae EMG

Both participants showed considerable asymmetry (i.e., magnitude of AUC) between the muscle activation level in LES-L3 and RES-L3 level while using their respective MPK devices. In contrast to the muscle activation trends observed with the MPKs, use of the PKA reduced the degree of asymmetry in muscle activation between the RES-L3 and LES-L3 (Figures 1A3,B3) during walking in both participants. There are three potential reasons that could have led to the reduction in muscle activation asymmetry while using the PKA. First, by virtue of the PKAs design, the energy provided by the active motors at the knee and ankle which propelled the ipsilateral side during the terminal stance and swing phases could have reduced the load on the erector spinae back muscles. Second, from the trends of the VGRF it can be seen that the PKA led to similar magnitude peak VGRF (i.e., F_Z1_/F_Z2_) as opposed to the MPKs. Third, it was observed that the activation level (AUC) of the contralateral RF muscle was considerably higher when ambulating with the MPK devices in comparison to the PKA for both the participants (Figures 1A4,B4). A similar muscle activation trend was seen for the contralateral MGC muscle activation in participant CS02. However, this MGC trend was subtle for CS01. These factors could have cumulatively facilitated the reduction of asymmetry in the lumbar L3 muscle activation pattern between the contralateral and the ipsilateral side while using the PKA. In contrast to the PKA, while using the MPK (passive energy) there is no power assist during the gait cycle.

This novel finding could hold implications for minimizing the chance of occurrence of low back injury and pain in transfemoral users over long term device use. This observation is significant because low back muscle activation and injury has been very scarcely studied in transfemoral prosthetic literature (Yoder et al., 2015; Shojaei et al., 2016). Asymmetry in lumbar erector spinae activation during gait is a typical muscle activation pattern in individuals with chronic low back pain (Cappozzo and Gazzani, 1982; Michaud et al., 2000; Lamoth et al., 2006a; Goujon-Pillet et al., 2008; Morgenroth et al., 2010; Hendershot and Nussbaum, 2013; Hendershot et al., 2013; Molina Rueda et al., 2013; Devan et al., 2014; Hendershot and Wolf, 2014; Shojaei et al., 2016). Indeed, such asymmetry has been linked to loss of mobility, debilitating quality of life and surgical interventions (Madeleine et al., 2008; Shojaei et al., 2016). It is highly possible that the muscle activation asymmetry, (Figures 1A3,B3) observed in the erector spinae at the L3 region while using the MPKs for locomotion could predispose these users to a higher risk of low back pain and injury in the future.

Based on this case series, we maintain that using the PKA led to more symmetric back muscle activation patterns for both our participants in comparison to their predicate MPKs.

This study is the first to systematically study lumbar muscle activation during different prosthesis use. Also, ours is the first study to report the lumbar muscle activation while using a PKA. From a clinical standpoint, the results observed have significant implications for consequences pertaining to return to work activities and the burden of long term costs. Further studies in this direction are warranted.

#### Variable cadence

Both users were able to achieve wider ranges of cadence while using the PKA (Figures 2A3,A5,B3,B5) on both the contralateral and the ipsilateral side. With a wide range of cadence, the PKA could offer improved potential in different walking environments over the predicate MPKs. Furthermore, it was observed that for most speed ranges higher than 0.4 m/s, the variability in stride time (CV%) was relatively higher while using the PKA than while using a MPK for both the contralateral and ipsilateral sides (Figures 2A3,A5,B3,B5). Indeed, in general, it is well-known that change in variability of movement and musculoskeletal injuries are related (Lamoth et al., 2006b; Madeleine et al., 2008; Lomond and Côté, 2010; Stergiou and Decker, 2011; Srinivasan and Mathiassen, 2012; Steele et al., 2014; Jayaraman et al., 2016). As far as this case study goes, it is too soon to comment if the higher variability that manifests while using the PKA is good or bad. However, unlike the MPK devices, the PKA device offers the clinician more control to fine-tune the leg parameters. To modulate gait variability. In general, changes in the variability of the movement that happen over time have been shown to be related to musculoskeletal injuries occurring due to repetitive movements. Only a structured longitudinal study focused on these outcomes can determine if such a feature is beneficial.

## Concluding remarks

This case series provide early stage results from a larger on-going clinical trial and thus are not broadly generalizable. However, the initial results from this ongoing trial of the PKA are promising for walking performance. Both users in this study were trained through just 12 sessions on the PKA and could perform as well as or in some cases better than MPK devices which the users have utilized full time for at least 2 years. In comparison to the MPKs, using the PKA led to more normalized knee and ankle kinematics, more normalized VGRF, and symmetric lumbar muscle activation at the erector spinae region. Additionally, CS01 showed better gait efficiency while using the PKA and CS02 demonstrated better endurance by achieving a higher walking speed. Based on these observations, we maintain that pursuing further research and development of such PKA devices for different terrains could potentially lead to the improvement of transfemoral prosthetic users mobility. The symmetric loading bear implications for minimizing the risk of secondary musculoskeletal injury occurring due to repetitive use. These findings hold potential implications for improving long-term device use and overall quality of life in transfemoral amputees.

## Author contributions

CJ, SD-D, SH-L and AJ: carried out the experimental activities, data analysis, participated in the design of the study, and drafted the manuscript; CM, MM, AN, and RS: carried out the experimental activities, data analysis, and drafting of the manuscript; BL, KG, and MG: assisted in the design of the study and in data collection. All authors approved the submitted version of the manuscript.

### Conflict of interest statement

MG and BL reports patents US 20130310949, US 9289317 B2, US 20130268090 A1, US 20150209159 A1, US 20120221119 A1 broadly relevant to this work. The other authors declare that the research was conducted in the absence of any commercial or financial relationships that could be construed as a potential conflict of interest.
