# Supplementary material for: Impact of Powered Knee-Ankle Prosthesis on Low Back Muscle Mechanics in Transfemoral Amputees: A Case Series
Source: Front Neurosci. 2018 Mar 22;12:134. doi: 10.3389/fnins.2018.00134 (PMC5874899; doi:10.3389/fnins.2018.00134)
Supplement: Supplementary file 1 [file DataSheet1.docx]

Supplementary Material

Impact of Powered Knee-Ankle Prosthesis on Low Back Muscle Mechanics in Transfemoral Amputees: A Case Series

Chandrasekaran  Jayaraman, Shenan Hoppe-Ludwig, Susan Deems-Dluhy , Matt McGuire, Chaithanya Mummidisetty, Rachel Siegal, Aileen Naef, Brian E Lawson, Michael Goldfarb, Keith E Gordon, Arun Jayaraman*

**Correspondence:** Arun Jayaraman [ajayaraman@sralab.org](mailto:ajayaraman@sralab.org)

# Supplementary Information

The third generation of the Vanderbilt Powered Prosthesis has been revised to incorporate several improvements. Similar to its predecessor ([1, 2]), it contains two brushless DC motors driving the knee and ankle joints through 3 stage backdrivable transmissions (a belt drive and two subsequent chain stages). For reliability and ease of fitting multiple subjects, all the electronics are contained in the shank, and the knee and ankle units are separated by a standard prosthetic pylon that can be adjusted for the user’s height. The embedded system measures joint kinematics, axial load in the shank, inertial signals, and estimates joint torque through monitoring of motor current all at an update rate of 500 Hz. Data are logged for tuning and assessment purposes on a micro SD card at the same rate. All sensors are of a non-contact variety for reliability and lifetime considerations, and there is three-fold redundancy in the joint kinematics measurements for fault detection. Similar to previous designs, a parallel spring is incorporated at the ankle to bias the motors torque capabilities and help with powered push-off [3]. For more details regarding the design of the prosthesis, please see [4].

[1] B. Lawson, H. A. Varol, A. Huff, E. Erdemir, and M. Goldfarb, “Control of Stair Ascent and Descent With a Powered Transfemoral Prosthesis,” *Neural Systems and Rehabilitation Engineering, IEEE Transactions on,* vol. 21, no. 3, pp. 466-473, 2013.

[2] B. Lawson, B. Ruhe, A. Shultz, and M. Goldfarb, “A Powered Prosthetic Intervention for Bilateral Transfemoral Amputees,” *Biomedical Engineering, IEEE Transactions on,* vol. PP, no. 99, pp. 1-1, 2014.

[3] F. Sup, H. A. Varol, J. Mitchell, T. J. Withrow, and M. Goldfarb, “Preliminary Evaluations of a Self-Contained Anthropomorphic Transfemoral Prosthesis,” *IEEE ASME Trans. Mechatron.,* vol. 14, no. 6, pp. 667-676, 2009.

[4] B. Lawson, J. Mitchell, D. Truex, A. Shultz, E. Ledoux, and M. Goldfarb, “A Robotic Leg Prosthesis: Design, Control, and Implementation,” *Robotics and Automation Magazine, IEEE,* vol. 21, no. 4, pp. 70-81, 2014.

# 2 Supplementary Figures and Tables

**2.1 Figures**

Predicate Device (MPK-1 (Genium) PKA Device


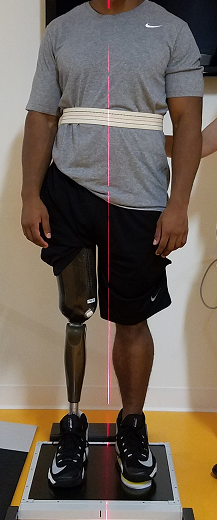

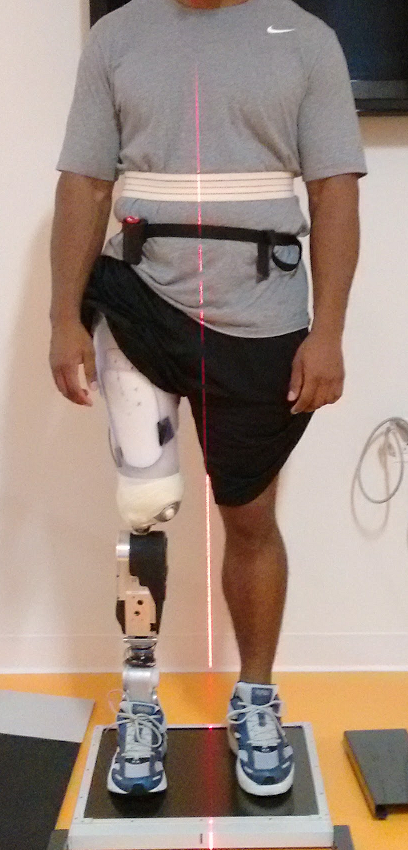


Figure S1. Participant CS01 with the MPK-1 (Genium) and the PKA device

Predicate Device (MPK-2 (Rheo-3) PKA Device


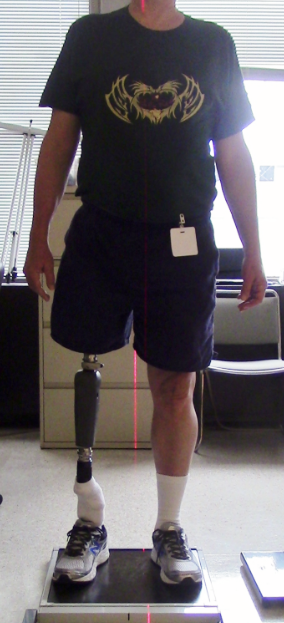

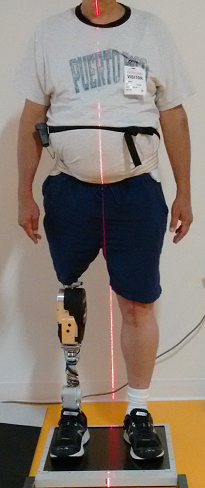


Figure S2. Participant CS02 with the MPK-2 (Rheo-3) and the PKA device

##
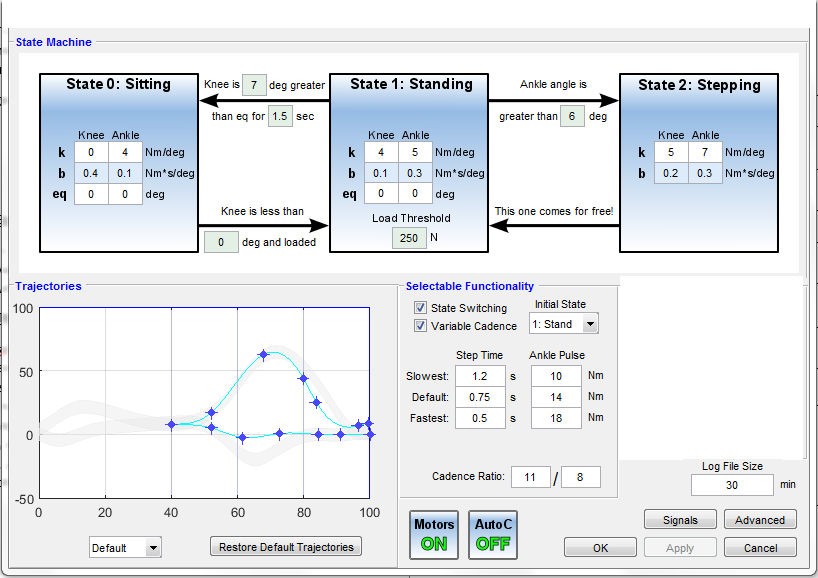


## Figure S3. CS01 Final PKA parameters. The finalized ankle pulse for different cadence can be seen in the ‘Ankle Pulse’ field in the ‘Selectable Functionality’ wizard. The swing phase reference trajectories for the knee and the ankle can be seen in the ‘Trajectories’ wizard.

##
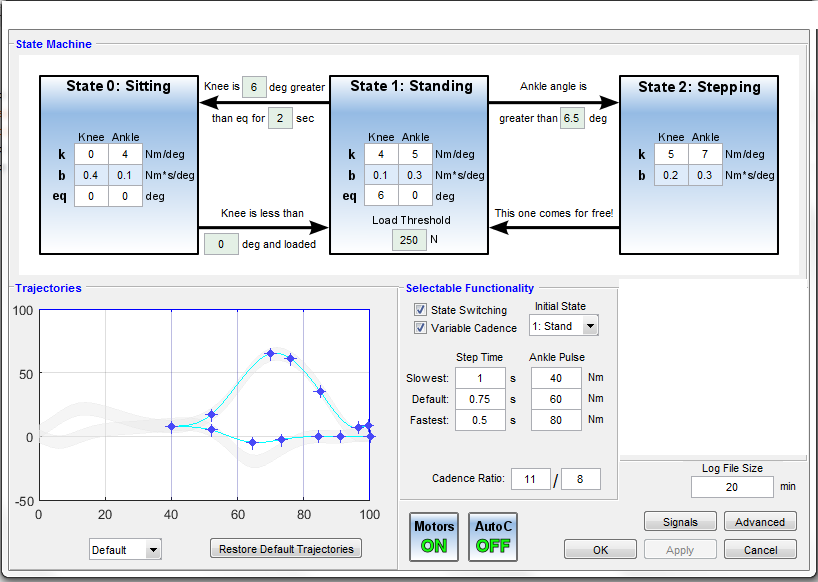


## Figure S4. CS02 Final PKA parameters. The finalized ankle pulse for different cadence can be seen in the ‘Ankle Pulse’ field in the ‘Selectable Functionality’ wizard. The swing phase reference trajectories for the knee and the ankle can be seen in the ‘Trajectories’ wizard.


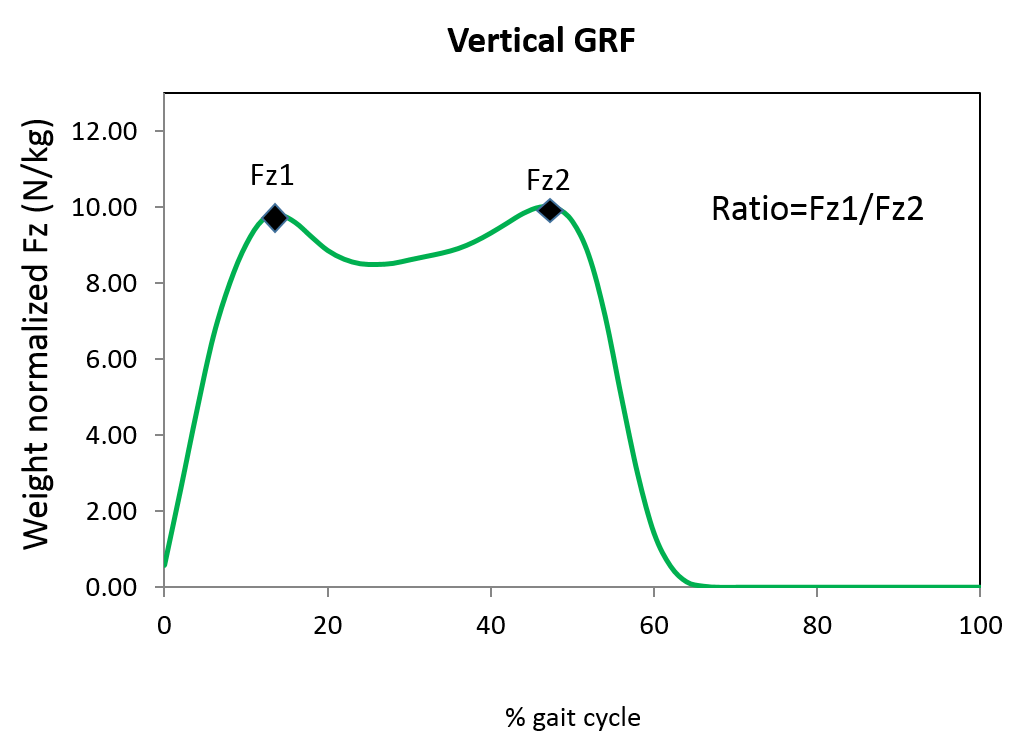


Figure S5. Calculation of the ratio of peak VGRF (Fz1/Fz2). Fz1 is the peak weight- normalized VGRF following the heel strike (F_Z1_ N/kg). Fz2 is peak weight-normalized VGRF at ankle push-off (F_Z2_ N/kg). Healthy control data from Winter 1991.

## 2.2 Tables

Table S1. Participant demographics and prosthetic device specifications

| **Subject** | *CS01* | *CS02* |
| --- | --- | --- |
| **Age** | 25 | 58 |
| **Sex** | M | M |
| **Height (m)** | 1.778 (5'10") | 1.905 (6'3") |
| **Weight (kg)** | 92.986 | 96.162 |
| **Cause of amputation** | Cancer | Infection s/p trauma |
| **Side affected** | Right | Right |
| **Years since amputation** | 11 | 44 |
| **Residual Limb Length** | Knee Disarticulation | Medium |
| **K level** | K4 | K3 |
| **Predicate Prosthetic Knee Component** | Otto Bock  Genium 3B1-2=ST | Ossur  Rheo 3 |
|  | 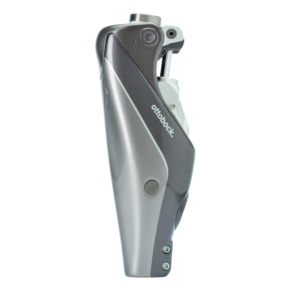  http://www.ottobockus.com/ | 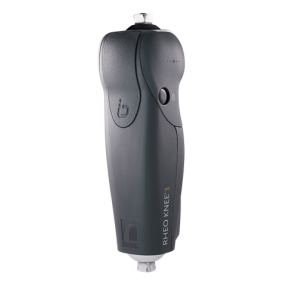https://www.ossur.com |
| **Prosthetic Knee Control** | Microprocessor | Microprocessor |
| **Prosthetic Knee Fluid Control** | Hydraulic Swing and Stance Control | Magnetic Rheologic Swing & Stance Control |
| **Mechanical Axis** | Single Axis | Single Axis |
| **Predicate Prosthetic Foot** | Multi-Axial Dynamic Response Carbon Fiber Foot | Multi-Axial Dynamic Response Carbon Fiber Foot |
| **Weight of Predicate Prosthesis (kg)** | 4.899 | 3.175 |

Table S2. Preferred Standing and Stepping Parameters for the PKA

| **Subject** |  | *CS01* | | *CS02* | |
| --- | --- | --- | --- | --- | --- |
|  |  | Knee | Ankle | Knee | Ankle |
| **State 1: Standing** | k (Nm/degree) | 4 | 5 | 4 | 5 |
|  | b (Nm*s/degree) | .1 | .3 | .1 | .3 |
|  | ϴ eq(degree) | 0 | 0 | 6 | 0 |
|  |  | | | | |
| **State 2: Stepping** | k (Nm/degree) | 5 | 7 | 5 | 7 |
|  | b (Nm*s/degree) | .2 | .3 | .2 | .3 |
|  |  | | | | |
| **Ankle Trigger Angle** | (degree) | 6 | | 6.5 | |
|  |  | | | | |
| **Step Time*** | Slowest (sec) | 1.2 | | 1.0 | |
|  | Default (sec) | .75 | | .75 | |
|  | Fastest (sec) | .5 | | .5 | |
|  |  | | | | |
| **Ankle Pulse (Push off Strength)*** | Slowest (Nm) | 10 | | 40 | |
|  | Default (Nm) | 14 | | 60 | |
|  | Fastest (Nm) | 18 | | 80 | |

*’Step Time’ refers to the peak period and the ‘Ankle Pulse’ refers of the unity-offset cosine torque pulse command that is superimposed with the trajectory controller. This pulse is a tunable parameter for the system. This value is not the net torque strength experienced by the user during push off.

Table S3. Benchmark activities

| **Item** | **Activities** |
| --- | --- |
| 1 | Independent donning/doffing prosthesis |
| 2 | Independent walking on level surfaces: indoors, tile and carpet transitions |
| 3 | Independent walking on level surfaces indoors with dual cognitive and/or motor task |
| 4 | Independent walking on level surfaces: indoors, with obstacles, starting/stopping |
| 5 | Independent in managing doors: able to open door, walk through, close door |
| 6 | Independent sit to/from stand transfers |
| 7 | Independent walking on level pavement, outdoors |
| 8 | Independent walking on uneven surfaces (grass, stone, uneven pavement), outdoors |
| 9 | Independent crossing streets |
| 10 | Independent treadmill walking |
